# Regular Exercise May Restore Certain Age-Related Alterations of Adaptive Immunity and Rebalance Immune Regulation

**DOI:** 10.3389/fimmu.2021.639308

**Published:** 2021-04-16

**Authors:** Gábor Papp, Krisztina Szabó, Ilona Jámbor, Marianna Mile, Alexandra Réka Berki, Attila Csaba Arany, Gabriella Makra, Peter Szodoray, Zoltán Csiki, László Balogh

**Affiliations:** ^1^ Division of Clinical Immunology, Faculty of Medicine, Institute of Internal Medicine, University of Debrecen, Debrecen, Hungary; ^2^ Institute of Sport Sciences, University of Debrecen, Debrecen, Hungary; ^3^ Department of Neurology, Faculty of Medicine, University of Debrecen, Debrecen, Hungary; ^4^ Department of Immunology, Oslo University Hospital, Rikshospitalet, Oslo, Norway

**Keywords:** aging, immunosenescence, physical activity, exercise, immune regulation

## Abstract

Age-related changes of the immune system lead to an increased morbidity and mortality due to enhanced vulnerability to infectious diseases and malignancies. Recent studies revealed the important effects of physical activity on immune functions, which may largely depend on the type of exercise, its intensity and duration. However, limited information is available regarding the immunological effects of sport activities in older ages. The aim of our study was to examine the changes in a wide spectrum of lymphocyte subtypes after regular workout among healthy elderly individuals. We enrolled 29 elderly women with sedentary lifestyle (mean age: 67.03 ± 3.74 years) to take part in a 6-week long functional conditioning gymnastic exercise program. The percentages of peripheral natural killer (NK), NKT cells, T and B lymphocyte subtypes (early-/late-activated T, naïve and memory T, cytotoxic T (Tc), T-helper (Th)1, Th2, Th17, T regulatory type 1 (Tr1), CD4^+^CD127^lo/-^CD25^bright^ Treg, as well as naïve and memory B cells) were determined by flow cytometry. Evaluation of the changes in functional capability of Treg cells was based on *in vitro* functional assays. At the end of exercise program, in parallel with improvements in body composition and physical performance, significant changes in naïve and memory lymphocyte ratios were observed. Importantly, levels of naïve Tc cells elevated, ratios of effector memory Tc cells decreased and distribution of memory B cells rearranged as well. Additionally, proportions of late-activated HLA-DR^+^ T cells increased, while percentages of anti-inflammatory interleukin (IL)-10 producing Tr1 cells, as well as immunosuppressive CD4^+^CD127^lo/-^CD25^bright^ Treg cells decreased following the exercise workout. Changes observed after the regular exercise program indicate an improvement in the age-related redistribution of certain naïve and memory cell proportions and a retuned immune regulation in older ages.

## Introduction

A properly functioning immune system is essential for the continuing survival of the host by maintaining a well-balanced defense against foreign organisms and protection from endogenous altered or virally transformed cells. However, old age inevitably leads to a number of changes that affect almost every element of the immune system and result in a progressive decline in immune functions. This age-associated process, called ‘immunosenescence’, disrupts the balance of immune homeostasis, consequently, elderly individuals become more susceptible to a wide range of infections, neoplasia and autoimmune diseases (1). The mechanisms of aging involve changes in both the innate and adaptive elements of the immune system, although alterations in the adaptive arm are more well-defined. Regarding innate immunity, we would refer to detailed and topical reviews available in this field (2, 3). Briefly, neutrophils and macrophages show decrease in their global functions involving reduced phagocyte functions and delayed cytokine secretions, while natural killer (NK) cells exhibit reduced cytotoxicity and diminished migration capacity, as well. Regarding the adaptive immunity, it has been previously observed that naïve T cells exhibit an absolute decrease with aging, which is accompanied with a relative increase in the population of various memory T cell types, primarily in effector memory T cells (4, 5). The diminished signaling of T-cell receptor (TCR) as well as insufficient expansion of CD4^+^ naïve T cells seems to be linked to age-related alteration in expression of microRNAs, namely the loss of miR-181 (6). Regarding CD8^+^ cytotoxic T (Tc) lymphocytes, numerous age-dependent changes were reported, including reduced cell numbers and impaired target binding, nevertheless killing capacity seems to remain intact upon successful binding (7, 8).

The changes associated with B lymphocytes are somewhat similar to the alterations observed for T cells. Naïve B cell proportion shows an age-related decrease presumably due to inadequate levels of B-cell activating factor (BAFF), which is a key maintenance factor for this lineage. On the contrary, memory B cells accumulate in older age and exhibit a more restricted repertoire of B cell antigen receptors (BCR) (9). Beside the significant reduction in B cell repertoire diversity, the decreased activation and proliferative capacity of B cells can also be observed in elderly individuals thus their functional responsiveness seems to diminish with aging. Animal models revealed that aging also affects the expression of activation-induced cytidine deaminase (AID), which is a key enzyme in class switching and somatic hypermutation (10). Consequently, the decreased functional affinity (avidity) of antibodies in older people leads to suboptimal antibody responses and impairs the quality and efficiency of antibody-mediated immune protection. Of note, a recent paper revealed that aging and obesity similarly impair antibody responses (11).

Interestingly, in older age, the intrinsic functional impairment of immune competence and the lack of proper immune defense are associated with a low-grade chronic systemic inflammation (12), that may be attenuated by regular physical exercises (13, 14), which are also common points with the immunological characteristics of obesity (15). Of note, studies in recent years have shown that obesity and insufficient physical activity have become a global health problem, and physical inactivity is the 4th most important risk factor for global mortality (16). There is growing evidence that regular exercise can reduce the development and progression of a numerous chronic diseases and disabling conditions, such as cardiovascular diseases, hypertension, osteoporosis, certain cancers, cognitive impairment, dementia and depression in the elderly (17). Regarding the immunological consequences of physical activity, regular but not strenuous exercise may increase the protective function of the immune system and reduce the risk of developing infections (18, 19). In contrast, high exercise training workloads are linked to immune dysfunctions and an increased risk for illness (20, 21). Consequently, physical activity has been shown to significantly affect immune functions; albeit, these effects seem to be highly dependent on the intensity and duration of different activities. However, the exact mechanisms of exercise-related immunological changes are still not known in detail and only limited information is available regarding the effects of sport activities on the adaptive immune system in older ages (22). Therefore, the aim of our study was to assess the changes in a wide spectrum of lymphocyte subtypes after a 6-week long, regularly performed, moderate-intensity functional training program in healthy elderly women.

## Materials and Methods

### Participants

Twenty-nine healthy elderly volunteers (female/male ratio: 29/0; mean age: 67.03 ± 3.74 years; mean BMI: 26.52 ± 1.89 kg/m^2^) living independently in the community were enrolled in the present study. Each volunteer completed a diet and physical questionnaire in the beginning and at the end of the study in order to assess their health condition and determine whether they comply with the criteria. Participants enrolled in the study were non-smokers, and they were abstaining from any physical exercises or sport activities, special diet and vitamin supplements for at least 3 months prior to the study. Moreover, exclusion criteria included ongoing viral or bacterial infection, allergic or autoimmune disease, chronic disease treated with continuous drug therapy, cancer; alcohol or drug addiction, psychiatric illness, insufficient compliance and dietary changes or usage of dietary supplements during the study period, as well.

### Training Protocol

All participants attended 60-minute long functional exercise sessions twice a week, in the morning (between 8 and 9 a.m.) for 6 weeks under stable climatic conditions (18 - 20°C) at UniFit Fitness and Gym Centre in Debrecen. We designed the exercise protocol according to the latest American College of Sports Medicine (ACSM) position stand on “Quantity and Quality of Exercise for Developing and Maintaining Cardiorespiratory, Musculoskeletal, and Neuromotor Fitness in Apparently Healthy Adults: Guidance for Prescribing Exercise” (23). All training sessions were supervised by trained instructors to minimize the risk of injury. Each session started with a 10 min warm-up section with treadmill (Star Trac Model 8TRx, Core Health & Fitness LLC, Vancouver, NE, USA), elliptical trainer (Star Trac Model 8CT, Core Health & Fitness LLC) or stationary bicycle (Activate Series Recumbent Lifecycle^®^ Exercise Bike, Life Fitness - Brunswick Corporation, Lake Forest, IL, USA). First, low intensity aerobic exercises were carried out and the intensity was enhanced up to 40-50% of maximum heart rate (HRmax) in order to protect the joints; thereafter, the intensity was increased up to 50-60% of HRmax for the next 20 min. Heart rate was monitored by the heart rate measurement system of the device. After the warm-up and aerobic sections, cyclic exercises were used to prepare the large muscles for muscle force development. Large muscle groups of the upper, lower limbs and core muscles were trained with the help of TRX, namely, TRX squat, TRX Low Rows, TRX push up, and TRX Standing Hip Drop exercises were carried out. Each exercise was set individually (angle of inclination was set between 10° and 45°) to allow the participant to execute 12 regular repetition intensively. Two participants were working together to get sufficient resting time. To improve the balancing ability, after the TRX exercises, participants were trained with Fitball for 10 min, during the first 3 weeks with help, while in the last 3 weeks alone - without help. All training was finished with stretching section. **Table 1** summarizes the detailed training protocol.

**Table 1:** Detailed training protocol.

Schedule	Exercises	Intensity level 1 (week 1-3)	Intensity level 2 (week 4-6)
10 mins	warm-up with treadmill/ellipse trainer/bicycle	40-50% HRmax	40-50% HRmax
20 mins	cyclic aerobic exercises with treadmill/ellipse trainer/bicycle	50-55% HRmax	55-60% HRmax
20 mins	TRX Squat	3 sets of 12-15 reps	–
	TRX Single Leg Squat	–	3 sets of 10 reps per foot
	TRX Low Rows	3 sets of 12-15 reps (range 10-45°)	–
	TRX Single Arm Low Rows	–	3 sets of 10 reps per arm (range 10-45°)
	TRX Push Up	3 sets of 12-15 reps (range 10-45°)	–
	TRX Push Up on one LEG	–	3 sets of 6 reps per leg (range 10-45°)
	TRX Standing Hip Drop	3 sets of 10 reps	3 sets of 15 reps
10 mins	Balance with Fitball/Bosu	in pairs with help	alone - without help
10 mins	Stretching	within normal range of motion	within maximum range of motion

HRmax, maximal heart rate.

### Physical Activity Assessment

Body composition analyses were carried out by an InBody 270 device (InBody, Seoul, South Korea). After the manual input of basic data such as height, sex and age, the measurements takes about 15 seconds. Besides the exact body weight, the analysis give a comprehensive picture on the mass of skeletal muscles and body fat regarding the whole body as well as its different parts including upper and lower extremities and trunk. Body Mass Indexes (BMI) were also calculated for each measurement.

Short Physical Performance Battery (SPPB) test was used for the evaluation of the severity of sarcopenia. SPPB evaluation includes a group of measures that combines the results of the gait speed, chair stand and balance tests (24). The scores range from 0 (worst performance) to 12 (best performance). CAMRY digital hand dynamometer (Camry Scale, South El Monte, CA, USA) was used to measure the changes in grip strength of both hands, values were given in kilogram.

### Blood Sampling and Analysis of Blood Cell Counts

For laboratory experiments, peripheral blood samples were taken baseline and 3 days after the 6-week long exercise program, in order to measure the immunological effects of the physical exercise program. All samples were collected between 8:00 and 9:00 a.m. to avoid circadian variation. Blood cell counts including total lymphocyte counts were analyzed from blood samples anticoagulated with ethylenediamine tetra‐acetic acid (EDTA) with ADVIA 2120i hematology system (Siemens, Munich, Germany).

### Determination of Lymphocyte Subpopulations According to Cell Surface Markers

For the comprehensive phenotypic analysis of peripheral lymphocyte populations, heparinized blood samples were collected from the healthy volunteers. The different cell populations were identified using fluorochrome-conjugated monoclonal antibodies against specific cell surface antigens. The basic characterization of lymphocytes performed by the combination of CD3-fluorescein isothiocyanate (FITC)/CD16+CD56-phycoerythrin (PE) (BD Biosciences, San Diego, CA, USA) and CD19-R-phycoerythrin-cyanine dye 5 (PE-Cy5) (Beckmann Coulter Inc., Brea, CA, USA) as well as CD4-FITC/CD8-R-PE/CD3-RPE-Cy5 (Dako Agilent Technologies, Santa Clara, CA, USA), monoclonal antibodies against cell surface markers. For the analysis of naïve and memory B cell subsets we used IgD-FITC, CD27-PE and CD19-PE-Cy5 (all from Beckman Coulter) antibodies, while the following monoclonal antibody combinations were used for phenotypic characterization of naïve and memory Th and Tc cells: CD45RA-FITC/CD4-PE (Beckman Coulter) and CD45RA-FITC/CD8-RPE (Bio-Rad Laboratories, Hercules, CA, USA), respectively, as well as CD62L-PE-Cy5 (Beckman Coulter). For the identification of early and late activated T cells we used CD69-PE-Cy5 (BD Biosciences) and human leukocyte antigen (HLA)-DR-PE (Bio-Rad) monoclonal antibodies, respectively, with the combination of CD3-FITC (Bio-Rad). We also investigated CD4^+^CD127^lo/-^CD25^bright^ Treg cells with the following reagents: CD4-FITC (BD Biosciences), CD127-PE and CD25-PE-Cy5 (both from Beckman Coulter). NKT cells were identified according to the combination of 6B11-PE and CD3-PerCP (both from BD Biosciences) monoclonal antibodies. IgG1-FITC/PE/PE-Cy5 (Beckman Coulter) isotype-matched antibodies were used in all procedures. For the identification of lymphocyte subpopulations in peripheral blood, freshly drawn (<3 h) anti-coagulated whole blood was used. After the incubation (30 minutes, at room temperature) with monoclonal antibodies, the hemolysis of erythrocytes was performed with 0.2% solution of formic acid, then cells were washed and fixed with 1% solution of paraformaldehyde and stored at 4°C until further measurement. The stained cells were assessed with Coulter FC500 flow cytometer (Beckman Coulter) and data were analyzed using Kaluza 1.2a software (Beckman Coulter).

### T Helper and T Cytotoxic Cell Identification With Intracellular Cytokine Analysis

For the identification of CD4^+^ Th cell subsets and CD8^+^ Tc cells, we used cytoplasmic cytokine staining method. Briefly, whole blood were diluted to 1:1 with saline solution and incubated with phorbol-12-myristate 13-acetate (PMA) (25 ng/ml), ionomycin (1 μg/ml) and Golgi Stop brefeldin A (10 μg/ml) (all from Sigma Aldrich, St. Louis, Missouri, USA) for 5 h at 37°C in 5% CO_2_ milieu. The following monoclonal antibodies were used for cell surface staining: CD4-PE-Cy5 or CD8-PE-Cy5 (both from Beckman Coulter). The cells were then fixed and permeabilized with Intraprep™ permeabilization reagent (Beckman Coulter) according to the manufacturer’s instructions, and intracellular cytokines were stained with the combination of interferon (IFN)-γ-FITC, interleukin (IL)-4-PE, IL-10-PE (all from BD Biosciences) and IL-17-PE (R&D Systems, Minneapolis, MN, USA) monoclonal antibodies. Measurements were performed and data were analyzed on Coulter FC500 flow cytometer (Beckman Coulter) equipped with Kaluza 1.2a software. IgG1-FITC (BD Biosciences) and IgG1-PE (R&D Systems) isotype-matched antibodies were used during the identification. Cells were quantified as their percentage in the CD4^+^ or CD8^+^ lymphocyte population.

### Suppression Assay of CD4^+^CD127^lo/-^CD25^bright^ Cells

The CD4^+^CD25^+^CD127^lo/–^ Regulatory T Cell Isolation Kit II (Miltenyi Biotech GmbH, Bergisch Gladbach, Germany) was used to obtain Treg cells, while with the CD4^+^ T Cell Isolation Kit (Miltenyi) we managed to gain effector Th cells from peripheral blood mononuclear cells (PBMCs) after Ficoll (Sigma-Aldrich, St Louis, MO, USA) gradients centrifugation. In case of Treg cells, the isolation was performed in a two-step procedure first using an LD, then an MS column according to the manufacturer’s instructions. The purity of isolated cell populations was above 98% in case of CD4^+^CD25^-^ effector Th cells and 95–98% regarding CD4^+^CD127^lo/-^CD25^+^ Treg cells. To carry out the suppressor activity assay, 8x10^4^ Treg cells were co-cultured with 8x10^4^ effector Th cells in the presence of human T cell activator CD3/CD28 microbeads (Thermo Fisher Scientific, Waltham, Massachusetts, USA) in 200 µl RPMI-1640 in a 96-well flat-bottom plate for 72 hours at 37°C in 5% CO_2_ condition. For controls and background measurements, Treg and effector Th cells were cultured separately as well. All measurements were performed in duplicate. Cell proliferation was measured by EZ4U colorimetric cell proliferation assay (Biomedica, Vienna, Austria) and the suppressor activity was detected by LabSystems 352 Multiskan MS Microplate Reader (Thermo Fisher Scientific) at 450/620 nm for optical density (OD) values, as described previously (25). The OD of mixed lymphocyte cultures (MLR) were corrected with the OD of Treg cells cultured separately as a background. The suppressor activity index was calculated as OD_Th effector_/(OD_MLR_-OD_Treg_).

### Statistical Analysis

Data were analyzed with GraphPad Prism 8 software (Graphpad Software, San Diego, USA). To assess the distribution of the data, Kolmogorov–Smirnov and Shapiro-Wilk normality tests were used. In case of Gaussian distribution, two-tail paired t test was used, on the other hand, if the data set differed from normal distribution, Wilcoxon test was performed. Differences were considered statistically significant at p < 0.05.

The number of enrolled individuals was estimated with *a priori* power calculation using G*Power v3.1.9.2. software (26). Data from previous studies with approximate changes in the percentages of different lymphocyte subpopulations during physical activity served as a basis to determine a medium effect size (Cohen’s *d_z_* = 0.6) (27, 28). Our calculation indicated that a sample size of at least 24 subjects were required to observe a statistically significant difference between matched data pairs with 0.8 statistical power at significance level of 0.05.

## Results

### Changes in Body Composition and Physical Performance

**Figure 1:**
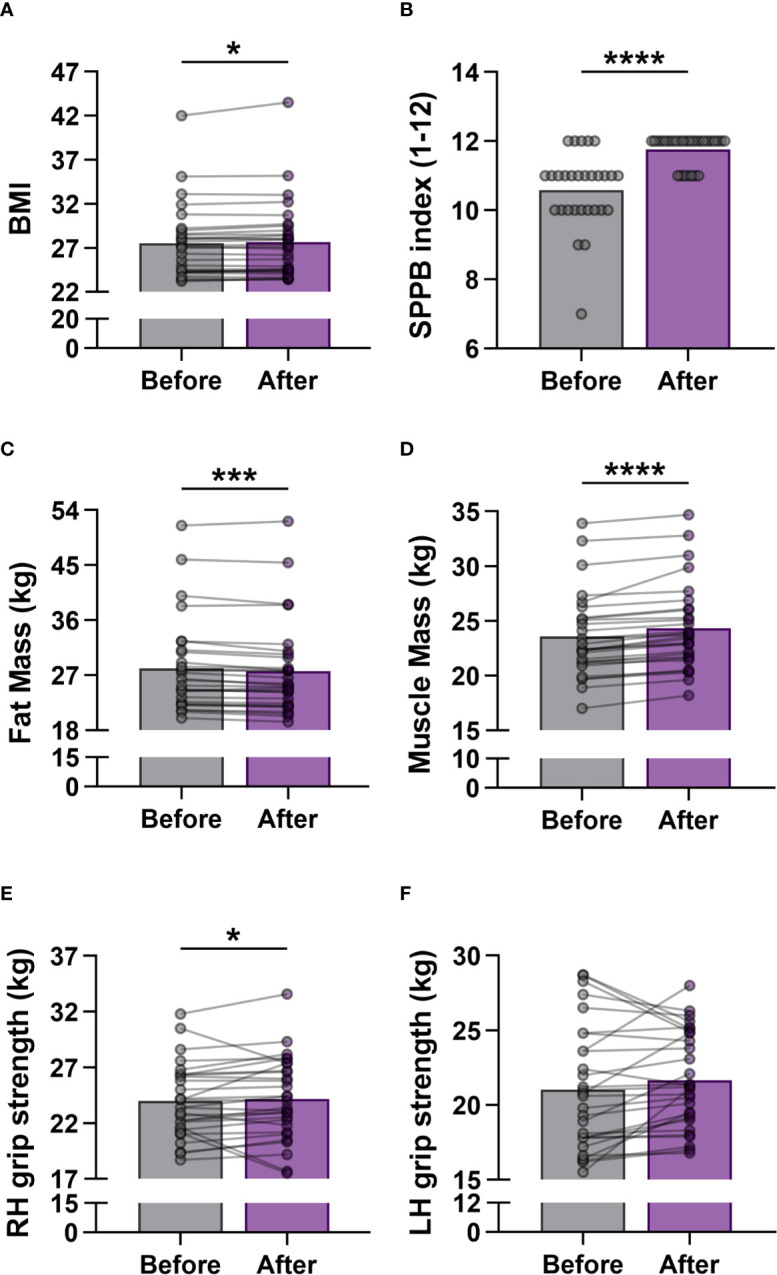
Measurement of physical activity in elderly women before and after the exercise training program. **(A)** Body Mass Index (BMI). **(B)** Short Physical Performance Battery (SPPB) index. The scores range from 0 (worst performance) to 12 (best performance). **(C)** Mass of body fat. **(D)** Mass of skeletal muscle. **(E)** Right hand (RH) grip strength. **(F)** Left hand (LH) grip strength. Values were calculated in kilogram (kg). Wilcoxon matched pairs test was used. Each data point represents an individual subject. Bars show the mean values. Statistically significant differences are indicated by *p < 0.05; ***p < 0.001; ****p < 0.0001.

In order to determine the effects of regular exercise on the fitness level of participants, measurements on body composition and physical performance were performed at baseline and repeated after the last exercise. A mild significant increase was observed between the before and after values of BMI (27.51 ± 4.132 vs. 27.66 ± 4.335; p = 0.018) (**Figure 1A**). The characteristics of body composition improved, the fat mass of the body decreased significantly (28.12 ± 7.556 vs. 27.69 ± 7.597; p = 0.0001), while the skeletal muscle mass of the body elevated significantly (23.57 ± 3.834 vs. 24.33 ± 3.907; p <0.0001) at the end of workout program (**Figures 1C, D**). The participants were also able to improve their physical performance after training, since SPPB scores were significantly increased (10.59 ± 1.086 vs. 11.76 ± 0.4355; p <0.0001) (**Figure 1B**), and the grip strength of hands (primarily the dominant one; 27 subjects were right-handed in the study group) were also enhanced (**Figures 1E, F**).

### Changes in the Distribution of Different Lymphocyte Subpopulations

**Figure 2:**
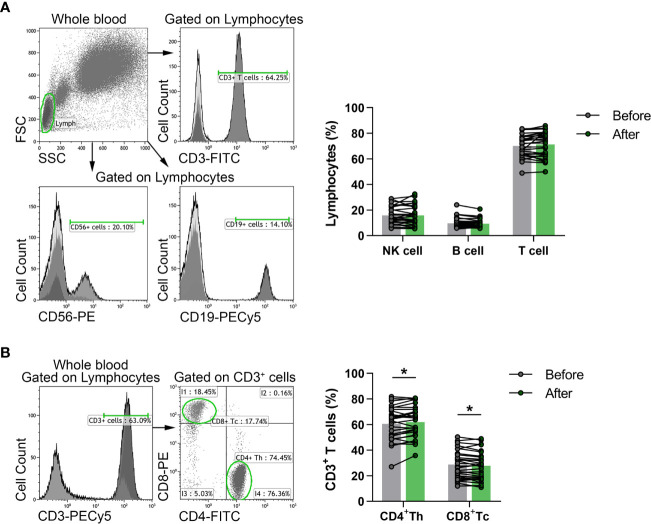
The distribution of peripheral lymphocyte subsets in elderly women before and after the exercise program. Whole blood of 29 participants were stained with fluorochrome-conjugated monoclonal antibodies as described previously. CD56^+^ NK, CD19^+^ B and CD3^+^ T cells were quantified as their percentage in the lymphocyte population, while CD4^+^ Th and CD8^+^ Tc cells were quantified as their frequencies in CD3^+^ cells. **(A)** Representative dot plots and histograms show the gating strategy of NK, B and T cell populations. The bar chart shows the percentages of NK, B and T cells. **(B)** Representative histogram shows the gating strategy and the dot plots demonstrate the distribution of Th and Tc cells. The bar chart indicates the frequencies of Th and Tc cells. Paired T test was used for statistical analysis. Each data point represents an individual subject, while bars show the mean values. Statistically significant differences are indicated by *p < 0.05.

Flow cytometric analyses were performed to evaluate the percentages of a wide-spectrum of immune-competent cell subsets in the peripheral blood of the participants. According to different cell surface antigens, listed in detail in the previous section, certain lymphocyte subpopulations, including NKT cells, Treg cells as well as activated, naïve and memory cell subsets were identified. Regarding the proportions of adaptive immune cells, flow cytometric evaluation included CD3^+^ T cells, CD3^-^CD56^+^CD16^+^ NK cells, CD19^+^ B cells, CD3^+^CD4^+^ Th cells and CD3^+^CD8^+^ Tc cells. However, considering the pronounced but transient exercise-induced changes in lymphocyte counts (29), we measured the absolute number of white blood cells in a group of healthy elderly volunteers (n = 15), and calculated total NK, T and B lymphocyte counts as well. Neither total lymphocyte counts nor total NK, T and B cell numbers showed changes 3 days after the last exercise session of the workout program (**Supplementary Figures 1A, B**). The percentages of NK cells, T and B cells in peripheral blood did not differ significantly either (**Figure 2A**); however, the distribution of T and B cell subsets showed fundamental changes. The frequencies of Th cells were significantly enhanced (60.469 ± 13.051 vs. 62.001 ± 12.105, p = 0.0177), while the ratio of Tc cells was significantly decreased (28.764 ± 10.888 vs. 27.697 ± 10.961; p = 0.0133) by the end of the exercise program (**Figure 2B**).

**Figure 3:**
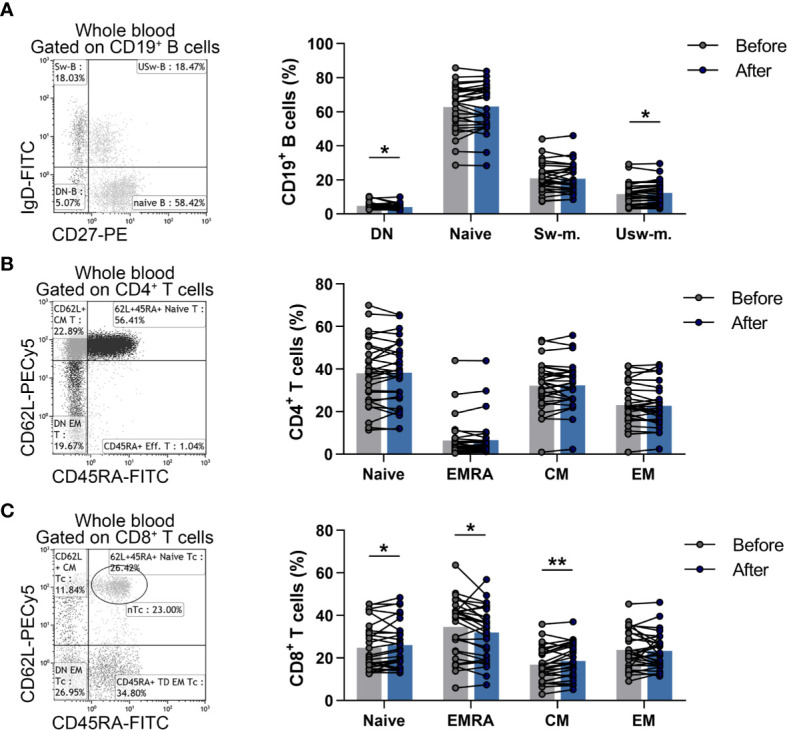
Changes in the division of naïve and memory lymphocyte subsets in elderly women after the workout program. Whole blood of 29 elderly individuals were stained with labeled monoclonal antibodies as described previously. B cell subsets were quantified as their proportions in CD19^+^ lymphocytes, Th subpopulations were assessed as their ratio in the CD4^+^ cells, while Tc cell subsets were quantified as their percentages in CD8^+^ cells. **(A)** Representative dot plot indicates the distribution of naïve, double negative (DN), switched and un-switched memory B cells. The bar chart shows the percentages of B cell subsets. **(B)** Representative dot plot demonstrates the distribution of naïve, effector memory RA^+^ (EMRA), central memory (CM) and effector memory (EM) Th cells. The bar chart indicates the frequencies of Th cell subpopulations. **(C)** Representative dot plot demonstrates the distribution of naïve, EMRA, CM and EM Tc cells. The bar chart indicates the frequencies of Tc cell subpopulations. Paired T test or Wilcoxon test was used for statistical analysis. Each data point represents an individual subject, while bars show the mean values. Statistically significant differences are indicated by *p < 0.05; **p < 0.01.

Naïve and memory B cells were identified as CD19^+^IgD^+^CD27^-^ naïve B cells, CD19^+^IgD^+^CD27^+^ un-switched memory B cells, CD19^+^IgD^-^CD27^+^ switched memory B cells, CD19^+^IgD^-^CD27^-^ double-negative (DN) B cells. We found that the ratio of DN B cells was significantly decreased (4.747 ± 2.042 vs. 4.020 ± 1.763; p = 0.0298) and the percentages of un-switched memory B cells were significantly heightened (11.619 ± 6.591 vs. 12.299 ± 6.524; p = 0.0246) compared to baseline values (**Figure 3A**).

Naïve and memory Th and Tc cells were referred to as CD4^+^CD45RA^+^CD62L^+^ naïve Th, CD4^+^CD45RA^-^CD62L^+^ central memory (CM) Th, CD4^+^CD45RA^-^CD62L^-^ effector memory (EM) Th, CD4^+^CD45RA^+^CD62L^-^ effector memory RA^+^ (EMRA) Th cells as well as CD8^+^CD45RA^+^CD62L^+^ naïve Tc, CD8^+^CD45RA^-^CD62L^+^ CM Tc, CD8^+^CD45RA^-^CD62L^-^ EM Tc, CD8^+^CD45RA^+^CD62L^-^ EMRA Tc cells. Although there was no significant difference in case of naïve and memory Th cell subsets (**Figure 3B**), a statistically significant elevation was found in the ratio of naïve Tc (24.773 ± 10.924 vs. 25.993 ± 10.828; p = 0.0408) and CM Tc (16.890 ± 7.972 vs. 18.593 ± 8.106; p = 0.0023) cells, while the percentages of effector memory Tc cells were decreased significantly (34.581 ± 13.372 vs. 32.081 ± 12.271; p = 0.0238) after the steady workout (**Figure 3C**).

**Figure 4:**
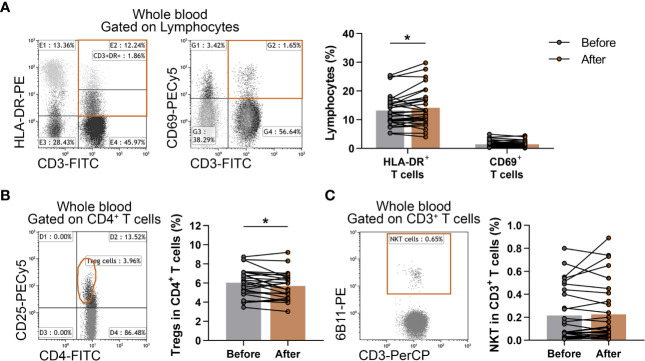
Assessment of the changes of activated T cells, Treg cells and NKT cells in elderly women after exercise program. Whole blood of 29 elderly women were stained with labeled monoclonal antibodies as described previously. Activated T cells were quantified as their percentages in the lymphocyte population, Treg cells were assessed as their frequencies in the CD4^+^ cells and NKT cells were quantified as their percentages in CD3^+^ cells. **(A)** Representative dot plots demonstrate the identification of CD3^+^HLA-DR^+^ late activated and CD3^+^CD69^+^ early-activated T cells. The bar chart indicates the frequencies of activated T cells. **(B)** Representative dot plot indicate the identification of CD4^+^CD25^bright^ Treg cells. The CD25^bright^ population was identified accurately after gating to CD127^lo/-^ T cells (not represented). The bar chart indicates the ratio of Treg cells. **(C)** Representative dot plot shows the determination and the bar chart indicates the frequencies of CD3^+^6B11^+^ NKT cells. Paired T test was used. Each data point represents an individual subject, while bars show the mean values. Statistically significant differences are indicated by *p < 0.05.

Additionally, CD3^+^CD69^+^ early-activated T lymphocytes, CD3^+^HLADR^+^ late-activated T lymphocytes, CD3^+^6B11^+^ NKT cells and CD4^+^CD127^lo/-^CD25^bright^ Treg cells were also determined. When we analyzed the ratio of activated T cells, we found that late-activated T cells differed significantly (13.189 ± 5.396 vs. 14.144 ± 6.547; p = 0.0412) from the baseline values (**Figure 4A**). Furthermore, the proportions of Treg cells showed a significant decrease after the workout program (6.043 ± 1.451 vs. 5.708 ± 1.430; p = 0.0370) (**Figure 4B**), but there was no significant difference in the percentages of NKT cells (**Figure 4C**).

### Peripheral T Helper Cell Subsets and Cytotoxic T Cells

**Figure 5:**
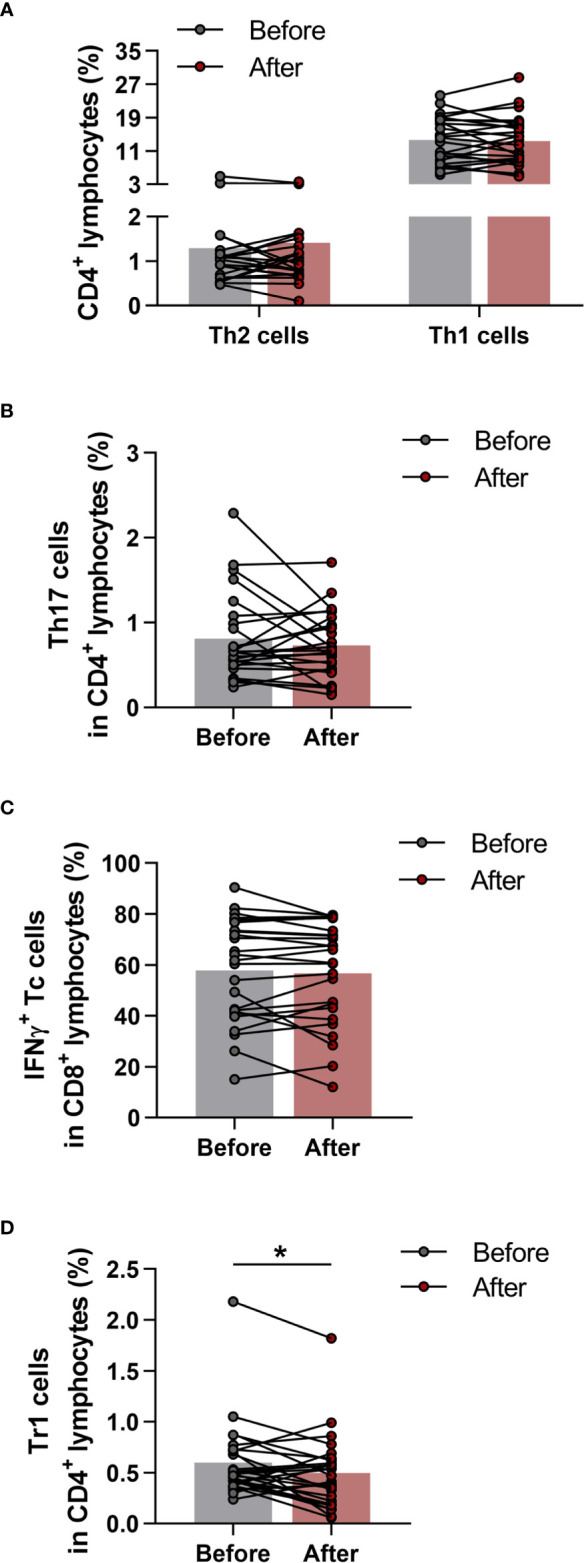
Determination of T helper and T cytotoxic cells with intracellular cytokine analysis in elderly women before and after the exercise program. Whole blood of 29 healthy participants were stimulated for 5h and dyed with fluorochrome-conjugated monoclonal antibodies with intracellular staining method as described previously. All cell subsets were quantified as their percentage in the CD4^+^ or CD8^+^ lymphocyte population. **(A)** Frequencies of IFNγ^+^ Th1 and IL-4^+^ Th2 cells. **(B)** Percentages of IL-17^+^ Th17 cells. **(C)** Ratio of IFNγ producing Tc cell. **(D)** Proportions of IL-10 producing type-1 regulatory (Tr1) cells. Paired T test was used. Each data point represents an individual subject, while bars show the mean values. Statistically significant differences are indicated by *p < 0.05.

The phenotypes within CD4^+^ cells were determined as CD4^+^IFN-γ^+^IL-4^-^ Th1 cells, CD4^+^IFN-γ^-^IL-4^+^ Th2 cells, CD4^+^IFN-γ^-^IL17^+^ Th17 cells and CD4^+^IL-10^+^ type-1 regulatory T (Tr1) cells. Cytotoxic T cells were identified as CD8^+^IFN-γ^+^ T cells. All cell subsets were quantified as their percentage in the CD4^+^ or CD8^+^ lymphocyte population. We found no significant differences in peripheral blood Th1, Th2, Th17 and Tc cells (**Figures 5A–C**), however, the ratio of Tr1 cells was significantly diminished (0.5989 ± 0.3724 vs. 0.4952 ± 0.3588; p = 0.0391) by the end of the 6-week exercise program (**Figure 5D**).

### Suppressor Function of CD4^+^CD127^lo/-^CD25^+^ Treg Cells

**Figure 6:**
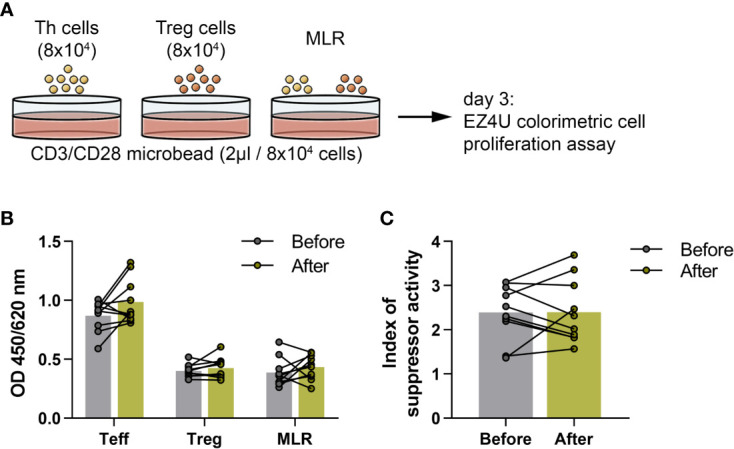
Suppressor activity of regulatory T cells in elderly women before and after the functional exercise program. **(A)** The schematic representation of the culture settings of the proliferations of CD4^+^CD25^-^ effector Th and CD4^+^CD127^lo/-^CD25^+^ Treg cells and mixed lymphocyte reaction (MLR) of Th and Treg cells in the presence of human T cell activator CD3/CD28 microbead for 72 hours. **(B)** The bar chart indicates the proliferations of Th, Treg cells and MLR. In the case of MLR, the Treg corrected optical density (OD) is displayed. **(C)** Suppressor activity index of Treg cells. Paired T test was used. Each data point represents an individual subject, while bars show the mean values.

We measured the functional activity of Treg cells obtained from 10 healthy elderly volunteers at the baseline and at the end of the training program. To investigate the suppressor activity of Treg cells, magnetically isolated CD4^+^CD25^-^ Th and CD4^+^CD127^lo/-^CD25^+^ Treg cells were cultured alone and together in the presence of human T cell activator anti-CD3/CD28 beads (**Figure 6A**). As expected, in the co-culture of MLR, the presence of Treg cells caused an obvious decrease in the proliferation, but there was no significant difference between the before and after values (**Figure 6B**). We found that the suppressor activity index of Treg cells did not differ significantly from the baseline values by the end of the exercise (**Figure 6C**).

## Discussion

Age-related changes in the immune system and their consequences became crucial issues lately, when human life expectancy keeps increasing. In many countries, the proportions of individuals aged 65 years or older are close or even above 20%; moreover, based on the present trends, by 2050, older people will represent approximately 25% of the population worldwide (30). However, in the absence of former evolutionary pressures, the human immune system was basically not designed to function for such a long lifetime, which results in the increased susceptibility to infections and cancers in the elderly (31). All these social and economic situation as well as individual health issues point to the urgent need to reveal effective interventions, which may delay the age-related alterations in immunity and prevent their pathological outcomes. Appropriate and regular physical activity is a promising candidate for this purpose. Nevertheless, physical training could be a double-edged sword that can improve or even worsen immune homeostasis. Exhaustive exercise leads to the reduction in proportions of immunocompetent cells with effector functions, as well as to a decrease of several cytokines, including IL-6, tumor necrosis factor (TNF)-α, IFN-γ, IL-1β, IL-2, IL-8 and IL-10, as well (21, 32). All of these deteriorating effects on immune functions may be further exacerbated by reducing the length of rest periods between the intensive physical exercises (33). In contrast, regularly performed moderate-intensity physical activity may contribute to a reduction in the risk of cardiovascular disease and cancer by stimulating immune functions (20). Based on the results of a previous study, physical activity may increase the number of NK cells and enhance the activity of neutrophil granulocytes in the peripheral blood (34, 35). Regarding the functions of the adaptive immune system, it seems that there is a significant difference between the acute impacts of single exercise bout and the effects of regular workouts. While a single exercise bout has no clear beneficial effect on antigen-specific B cell responses to influenza and pneumococcal vaccination (36, 37), numerous studies demonstrated higher antibody responses to influenza vaccination in older adults, typically over 60 years of age, who regularly undertook moderate or vigorous exercise training compared to inactive controls showing that the immunological effects of regular workout may be particularly beneficial in enhancing otherwise poor responses in older age (38, 39). In addition to the intensity and regularity of exercise, the duration of a training session is also crucial for direction of early consequences on immune system. In non-professional athletes, a short exercise is associated with an increase in plasma levels of anti-inflammatory cytokines such as IL-4 and IL-10; however, a longer exercise results in an increase of levels of pro-inflammatory cytokines such as IL-1β and TNF-α (40).

In our present study, besides examining the improvements in physical conditions, we focused on the immunological changes in healthy elderly women performing a 6-week training program with moderate-intensity. Due to the regular physical exercises the characteristics of body composition improved, the fat mass decreased, while skeletal muscle mass increased significantly, and the latter led mild increase in BMI value. In parallel with alteration in skeletal muscle mass, muscular strength also increased which reflects a significant improvement in the physical performance of the elderly individuals.

Importantly, changes induced by intense physical exercise may last at least 24 hours, and even moderate acute exercise induces significant immune alterations for several hours (29), therefore we carried out the laboratory measurements 3 days after the last workout. Based on our observations, neither total lymphocyte counts nor total NK, T and B cell numbers showed any alterations 3 days after the last exercise session of the workout program indicating no distorting effects of transient immunological changes, including post-exercise lymphocytopenia.

Focusing on the distribution of naïve and memory subpopulations of B cells, we revealed that the ratio of DN B cells decreased, while percentages of un-switched memory B cells increased at the end of workout program. Of note, the generation of DN B cells seems to be a typical phenomenon due to the aging of immune system. These cells may be exhausted memory B cells that have reverted their expression of CD27 as they have switched the heavy chain of immunoglobulin. Former studies suggest that these cells do not express HLA-DR, CD80 and CD40 which are important molecules for antigen presentation and T-B cooperation (41). Among memory B cells, IgD^+^CD27^+^ or un-switched memory B cells are committed to polyclonal B-cell responses to thymus-independent antigens and they are important in defense against extracellular bacterial pathogens. It has been demonstrated, that IgD^+^IgM^+^CD27^+^ or so called IgM memory B cells decreased excessively in elderly people, besides this decline could be involved in the increased susceptibility of the elderly to pneumococcal disease (42). Considering the aforementioned observations, the decreased ratio of DN B cells with increased percentages of un-switched memory B cells suggest a beneficial rearrangement in the distribution of memory B cell subsets in older ages after regular exercises.

As for T cell subtypes, we revealed an increase in HLA-DR^+^ late-activated T cell percentages, which may indicate a more activated immunological state of the elderly individuals at the end of the 6-week long training program. It has already confirmed that the ratio of HLA-DR^+^ cells in CD4 and CD8 lymphocytes are increased in elderly individuals compared to young adults, however it is more pronounced in CD8 than in CD4 T cells. In vitro stimulation of both subset revealed that activated CD8 T cells gained more HLA-DR expression during culture (27). Therefore, we believe that the observed increase in the expression of HLA-DR was not solely the result of the elevation in total CD4^+^ Th cell proportions after the training program. Interestingly, recent studies suggested that HLA-DR^+^CD8^+^ T cells represent natural regulatory CD8^+^ T cells and most frequently express high level of CD28 and low level of CD45RA. The ratio of these regulatory CD8^+^ T cells is increased in elderly individuals, however their inhibitory molecules and regulatory function are decreased in spite of their elevated cell numbers (43, 44). Nevertheless, to get a better view on the activation state of T cells, further studies are needed on the different T subsets including naïve and memory Tc and Th cells, as well.

In our study, total CD8^+^ Tc cell levels decreased and the distribution of CD8^+^ Tc cell subsets showed significant changes at the end of workout program. These results are in accordance with previous observations. Age-associated changes has been described to occur more profoundly in peripheral blood CD8^+^ Tc cell population than CD4^+^ Th cells (45). Regarding the whole CD8^+^ Tc cell compartment, a former study reported an increase in their proportion during both normal- and high-intensity trainings; however, its percentage fell below baseline levels after the exercise (46). Recently, several studies investigated the mobilization of CD8^+^ T cell subsets during exercise and revealed that late-differentiated or RA^+^ effector memory Tc subset is exclusively decreased post-exercise period (47). It is established that this subpopulation expresses elevated β2-adrenergic receptor density and sensitivity which could explain their higher responsiveness to exercise, and it could be further augmented by cytomegalovirus seropositivity (27, 48). Of note, the expression of a broad number of markers overlaps or associates with each other and results in the large heterogeneity of CD8^+^ T cells. Accordingly, several studies suggest that CD62L^-^CD45RA^+^ T_EMRA_ cells rather show highly functional capacity than an acutely activated phenotype since these antigen-experienced subset gradually down-regulate the expression of several activation and co-stimulatory markers including CD27, CD38 and HLA-DR while produce high level of inflammatory IFN-γ cytokine and cytotoxic enzymes (27, 49–52). This late-differentiated effector phase has multiple characteristics of senescence, including the loss of proliferative capacity which could be measured by the increase of CD57 expression, the acquisition of typical markers of the NK cells such as CD85j, CD158b/j, KLRG1 and short telomere length (27, 51–54). Moreover, these exercise-sensitive RA^+^ effector-memory Tc cells are largely negative for skin specific cutaneous lymphocyte-associated antigen (CLA), thus their migration from the circulation could rather be directed by CXCR3 and CCR5 chemokines or site-specific homing molecules like α4β7 integrin toward tissues, including lungs or Peyer’s patches (55). Importantly, in our present study, elderly individuals exhibited lower proportions of late-differentiated effector memory CD8^+^ T cells and higher proportions of naïve CD8^+^ T cells in blood samples at the end of the workout program. This exercise-associated shift has been reported in a recent study, in which intensive and moderate training resulted in the marked reduction of T_EMRA_ cells, moreover they showed that the training-lifestyle was associated with telomere length preservation. The concept is that reduced telomere erosion are associated with better immune response against infectious agents (47). Considering that aging is associated with a decline in the number and proportion of naïve T cells, and an accumulation of memory T cells with limited specificities (4), the applied workout intervention may be effective for the rearrangement of these cell proportions. Although the exact mechanisms behind these anti-immunosenescence effects of regular exercise are not elucidated yet, a possible theory may answer some questions. It is assumed that the homeostatic number of peripheral T cell repertoire is tightly regulated by a negative-positive feedback mechanism that involves IL-7 and the thymus. Several studies discussed a theory that the “space” of naive T cells are used up by senescent T cells over a lifetime and the elevated number of late-differentiated T cells inhibit the generation of new naive T cells and accelerate thymic involution (56). However, regularly executed moderate exercise might prevent immunosenescene by decreasing the accumulation of T_EMRA_ cell clones by their mobilization into the circulation and subsequent extravasation to peripheral tissues where they are exposed to H_2_O_2_ induced apoptosis. Thereby, the negative feedback is reserved and the positive feedback increases thymic output and the accumulation of antigen-inexperienced naive T cells at the periphery (57, 58).

We also observed an increase in total CD4^+^ Th cell proportions; nevertheless, the regular exercises did not lead to a change in Th1:Th2 ratio in the elderly population. Importantly, it is known that aging is associated with a predominance of Th2 cells, whereas there is a decline in Th1 cell proportions among T-helper cell population (59). Similarly, strenuous physical activity induces a shift in Th1/Th2 balance to Th2 cell predominance (60, 61). In our investigation, the exercise interventions with moderate intensity did not lead to further shift in Th1:Th2 ratios, which change would have been an adverse effect in older age. Moreover, we did not observe any alterations in the ratios of naïve and memory Th subsets either. Based on our observations, proportions of Th subsets may hardly influenced by exercise in older ages.

Regarding T cell subsets with immunoregulatory functions, it was reported that acute, high intensity exercises may cause a significant elevation in Treg cells (62), and recently, higher levels of TGF-β, which is a known anti-inflammatory cytokine that contributes to the immunosuppressive effects of Treg cells, were also reported after workout (63). On the contrary, a recent study reported a decrease in peripheral Treg cell numbers after regular exercises (64). Based on our observations, the levels of immunosuppressive IL-10-producing Tr1 cells as well as CD4^+^CD127^lo/-^CD25^bright^ Treg cells showed a significant decrease in the elderly individuals after completing the 6-week long functional exercise program. These observations shed light on the probably most important effects of physical exercises and sports on immune regulation in older ages. Of note, at the level of adaptive immune system, the network of regulatory T cells is primarily responsible for the suppression of the effector functions of immune responses. Among others, the two major representatives of this system are denoted as induced Treg cells (iTreg) and CD4+CD25^bright^FoxP3^+^ natural Treg cells. Although, in the regulation of peripheral T cell immune responses, several types of iTreg cells participate, its most widely investigated, key subset is the IL-10-producing Tr1 cell (65). Frequencies of Treg cells also alter with age, with Treg numbers increasing (66, 67), which is considered to contribute to immune suppression in old age (68). Our present findings on the effects of exercises on Treg proportions might point out the most crucial consequence of physical activity in older ages. Additionally, beside quantitative changes, we also investigated qualitative changes in Treg cells. Based on the results of the *in vitro* functional tests examining the suppressor capability of Treg cells, neither enhanced nor impaired function was observed. Taken together, the exercise-associated decrease in the proportion of Treg cells does not trigger a counter-regulatory mechanism that would lead to an enhancement in their suppressor activity. On the other hand, the lower Treg number is not accompanied with a decline of their suppressor function, which impairment would potentially increase the risk for developing autoimmune disorders or allergic diseases (65, 69).

Our findings suggest that exercise-induced changes in the distribution of certain naïve and memory B and T cell subsets as well as in the proportions of regulatory T cells presumably indicate a retuned immune regulation and a restored responsiveness of the immune system. Thereby, regular exercise, besides improving physical condition and age-related sarcopenia, may also delay or even reverse immunosenescence therefore can be particularly beneficial in maintaining appropriate immune functions in older ages.

## Data Availability Statement

The original contributions presented in the study are included in the article/**Supplementary Material**. Further inquiries can be directed to the corresponding author.

## Ethics Statement

The studies involving human participants were reviewed and approved by the Ethics Committee of the University of Debrecen (protocol number: 4839-2017) and the Policy Administration Services of Public Health of the Government Office (registration number: 25040-4/2017/EÜIG). All experiments carried out were in compliance with the Declaration of Helsinki. The patients/participants provided their written informed consent to participate in this study.

## Author Contributions

GP designed the study, supervised the research program, analyzed data, and wrote the main manuscript text. KS performed laboratory experiments, analyses data, prepared figures, and contributed to manuscript writing. IJ performed laboratory experiments. AB and AA contributed to enrolling participants and data collection. GM instructed exercise sessions. MM designed and supervised the workout program. ZC and PS contributed to the interpretation of findings and edited the manuscript. LB provided conceptual advices and contributed to the final version of the manuscript. All authors contributed to the article and approved the submitted version.

## Funding

The research was supported by the GINOP-2.3.2-15-2016-00062 project, which was co-financed by the European Union from the European Regional Development Fund. The work of GP was supported by the János Bolyai Research Scholarship of the Hungarian Academy of Sciences and the ÚNKP-20-5 New National Excellence Program of the Ministry for Innovation and Technology. AA received scholarship from the talent development program of EFOP-3.6.1-16-2016-00022 project co-financed by the European Union and the European Social Fund.

## Conflict of Interest

The authors declare that the research was conducted in the absence of any commercial or financial relationships that could be construed as a potential conflict of interest.
