# Supplementary material for: Regular Exercise May Restore Certain Age-Related Alterations of Adaptive Immunity and Rebalance Immune Regulation
Source: Front Immunol. 2021 Apr 16;12:639308. doi: 10.3389/fimmu.2021.639308 (PMC8085426; doi:10.3389/fimmu.2021.639308)
Supplement: Supplementary file 1 [file Table_1.docx]

Supplementary Material


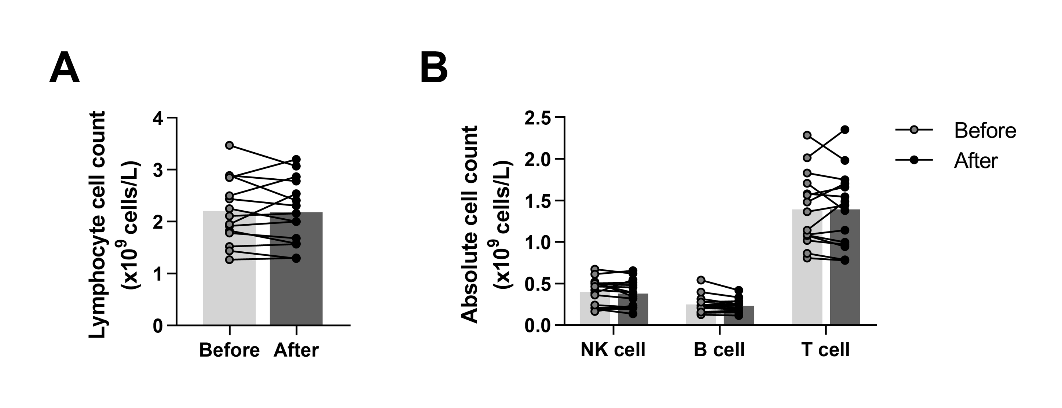


**Supplementary Figure 1.** **The distribution of the total lymphocyte cell count and absolute numbers of peripheral lymphocyte subsets in elderly women before and after the exercise program.** Blood cell counts including total lymphocyte counts were analysed with ADVIA 2120i hematology system (Siemens, Munich, Germany) at the Department of Laboratory Medicine, Faculty of Medicine, University of Debrecen. Based on the lymphocyte counts and the percentages of main cell subsets measured in the study by flow cytometry, absolute cell numbers were calculated using the following calculation: absolute CD3^+^ T cells (10^9^/L) = lymphocytes count (10^9^/L) x (percent CD3^+^ T cells / 100). (**A**) Total lymphocyte cell count before (baseline) and after (6 weeks later) the training program. (**B**) Absolute numbers of CD56^+^ NK cells, CD3^+^ T cells and CD19^+^ B cells. Each data point represents an individual subject, while bars show the mean values.
